# Concepts for point-of-care ultrasound training in low resource settings: a scoping review

**DOI:** 10.1186/s13089-025-00427-3

**Published:** 2025-05-15

**Authors:** Friedrich Eppel, Friederike Hunstig, Sabine Bélard, Benno Kreuels

**Affiliations:** 1 Institute of International Health, Charité, Universitätsmedizin Berlin, Berlin, Germany; 2 Department of Emergency Medicine, Klinik Floridsdorf, Vienna, Austria; 3 Research Group Neglected Diseases and Envenoming, Bernhard Nocht Institute for Tropical Medicine, Hamburg, Germany; 4 Institute of Tropical Medicine, University of Tübingen, Wilhelmstr. 27, 72074 Tübingen, Germany; 5 German Center for Infection Research (DZIF), Partner Site Tübingen, Tübingen, Germany; 6 Department of Medicine, School of Medicine, Kamuzu University of Health Sciences, Blantyre, Malawi; 7 Section for Tropical Medicine, I. Department of Medicine, University Medical Center Hamburg-Eppendorf, Hamburg, Germany

**Keywords:** Point of care ultrasound, POCUS, Training, LMIC

## Abstract

**Background:**

Point-of-care ultrasound (POCUS) is a potent diagnostic tool especially in resource-limited settings. The implementation of POCUS diagnostics requires adequate training of POCUS operators. This scoping review aimed to identify and describe POCUS training concepts that have been applied in low-and middle-income countries (LMICs).

**Methods and findings:**

All studies on diagnostic POCUS training in LMICs that could be found in the Cochrane, Embase, Google Scholar, and Medline databases up to July 6, 2023, were included and data was extracted for descriptive analysis. The review protocol was registered at OSF https://doi.org/10.17605/OSF.IO/8FQJW. A total of 53 publications were included with 59% of studies (n = 31) conducted in Africa and 23% (n = 12) in Asia. The majority of studies (n = 41, 81%) described short courses amongst which 40% were one-off sessions and 60% described longitudinal trainings. Curricula were mostly related to emergency medicine and obstetrics and organ-focused protocols (lung n = 29 (54%), cardiac n = 28 (53%), obstetric n = 23 (43%)). Trainees were largely medical doctors and clinical officers with minimal or absent ultrasound skills. Training challenges included resource constraints and lack of context adaptation. Best practice recommendations included focus on hands-on training, low trainer to trainee ratio, protected training time, online training options, use of local trainers, short and concise training manuals in print, continuous supervision and early and on-going evaluation, as well as tele-mentoring.

**Conclusions:**

Context integration and focus on local needs, trainer availability and suitability, durable equipment and maintenance, as well as emphasis on hands on training including patients with relevant pathology, were key aspects for targeted and sustainable POCUS training in LMICs identified in this review.

**Supplementary Information:**

The online version contains supplementary material available at 10.1186/s13089-025-00427-3.

## Introduction

Point-of-care ultrasound (POCUS) is a diagnostic tool that is increasingly being demonstrated to be of significant value in the front-line evaluation and management of a range of clinical conditions across various medical fields, including obstetrics, emergency medicine, and infectious diseases in low- and middle-income countries (LMIC) [1–7].

The “diagnostic gap” has been highlighted as *“the biggest gap in the cascade of care across all conditions, across all settings”* [8]. Consequently, POCUS has been proposed as an essential diagnostic tool at the primary healthcare level to help close this gap [8, 9]. Nevertheless, only a minority of primary care facilities in non-high-income countries are currently equipped to provide basic ultrasound diagnostics [10].

In comparison to other cross-sectional imaging modalities, POCUS has several advantages. These include lower cost, the availability of small portable devices, and the absence of radiation protection requirements [9]. A recent review by Abrokwa et al. emphasized the potential of POCUS to enhance patient management by non-specialist front-line healthcare providers in LMICs. The concept of “task-shifting” POCUS represents a promising approach to address the shortage of specialists [6].

The implementation of POCUS requires the provision of appropriate training for POCUS operators [9], which is associated with a number of challenges. These include the high costs involved, limited infrastructure in geographic and academic isolation, as well as the potential for distinct disease spectra, and the need for sustainability [6, 11, 12]

The aim of this review was to identify POCUS training concepts in LMICs and to reveal challenges associated with the conduct of POCUS training in these settings. Understanding POCUS training approaches and their challenges can guide the design of future POCUS training programs that will be required, to roll out access to POCUS diagnostics in LMICs.

## Methods

The primary objective of the review was to identify and describe the training concepts employed in POCUS training programs in LMICs. The secondary objective was to compare identified POCUS training concepts in relation to POCUS quality, sustainability, scalability and medical condition.

We conducted a scoping review in accordance with the *PRISMA Extension for Scoping Reviews (PRISMA-ScR)* guidelines and checklists [13] and the recommendations of *JBI’s Manual for Evidence Synthesis on Scoping Reviews* [14] as well as the *Updated methodological guidance for the conduct of scoping reviews* [15].

The review protocol for this research was registered on November 21st, 2022 with Open Science Framework (OSF) with the 10.17605/OSF.IO/8FQJW. As published and publicly available literature forms the basis of the review, ethical approval was not required for this investigation.

### Database search

A search of the electronic databases Medline, Embase, Cochrane, and Google Scholar was conducted on December 8th, 2022 with an update performed on July 6th, 2023. The search strategy was formulated using the Participants/Population-Concept-Context (PCC) method [16] and centered around the three main themes: point-of-care ultrasound, training and low- and middle income countries. The terms “point of care ultrasound”, “POCUS”, “bedside ultrasound”, “point of care sonography”, “bedside sonography” or “bedside echocardiography” were used as synonyms. LMICs were listed in accordance with the World Bank Definition from 2022 [17]. Additionally, the terms “low income country”, “middle income country”, “developing country” as well as “resource limited setting” were employed as synonyms. Alternative search terms for “training” were “education” or “course”. The complete search strategy for Pubmed/Medline can be found in the supplementary material (Supplement 1). The search strategy was modified for the Embase and Cochrane databases using the “Polyglot Search Translator”, a tool designed to facilitate the translation of search syntax between different databases [18].

### Eligibility criteria

In accordance with the PCC method, the participants/population were defined as all frontline healthcare providers not specialized in imaging, utilizing or intending to utilize POCUS. Publications focusing solely on providers with specialized expertise in imaging, such as radiologists, echo specialists, specialized obstetricians and professional imaging technicians were excluded from this review. The concept was defined as POCUS training, where POCUS is understood as a diagnostic method that enables clinicians to answer simple and specific clinical questions at the bedside with the aim to guide clinical management. For the purposes of this review POCUS training was defined as any formal teaching or learning activity with the objective of enabling health care providers to perform POCUS and/or to enhance their POCUS performance. The context under investigation was all POCUS training settings in LMICs, including on the job training settings as well as distant learning and simulator-based settings. Studies on advanced ultrasound training (not POCUS) aimed solely at specialized or specializing health care providers (like radiologists, echocardiographers, professional imagers) were excluded. Moreover, publications on the training of POCUS providers in high income countries as well as publications on the training of exclusively procedural ultrasound were excluded. The review included published literature containing original data. No restrictions were placed on the year of publications. No language restrictions were applied during the search and screening process. However, one potentially eligible study published in Chinese was excluded due to the lack of a reliable translation for data extraction [19].

### Data methodology

Publications were organized in Endnote 20 [20]. Title, abstract and full-text screening were conducted using Rayyan QCR, a web application designed for the management of systematic reviews [21]. Following the removal of duplicates, two independent reviewers (FE, FH) conducted title and abstract screening in accordance with the pre-defined selection criteria. Subsequently two independent reviewers (FE, FH) undertook a full text screening of all eligible publications. Discrepancies were resolved through discussion with two further reviewers (SB, BK). A comparison of the data extracted by the two reviewers (FE, FH) from a random sample of 10% of the total material yielded no discrepancies. Consequently, the remaining 90% of publications were subjected to data extraction by one reviewer (FE or FH) and subsequently verified by the other reviewer (FE or FH). A comprehensive pre-specified dataset including the following elements was extracted: authors, title, year of publication, study design, country, target clinical specialty, study objective, trainee and trainers’ details, duration of training, training content, mode of delivery, ultrasound devices used, training challenges, evaluation methods, evaluation challenges, key findings, limitations. The data was organized and analysed using Microsoft Excel (Version 16.66.1) and presented in a descriptive manner with the use of text and tables.

### Quality assessment of studies

To assess the methodological quality of the included studies a five-question critical appraisal tool was developed for this study, based on previously published tools [22, 23]. Each question could be answered with one of the following options: yes/partly/no/not applicable. Different numerical scores were assigned to each answer (yes = 2 points, partly = 1 point, no = 0 points). A score of 9–10 was deemed to indicate a high level of methodological quality, 7–8 was considered to indicate a moderate level of methodological quality, and a score of less than 7 points was considered to indicate a limited level of methodological quality. The complete tool can be accessed in the supplementary material (Supplement 2).

## Results

**Fig. 1:**
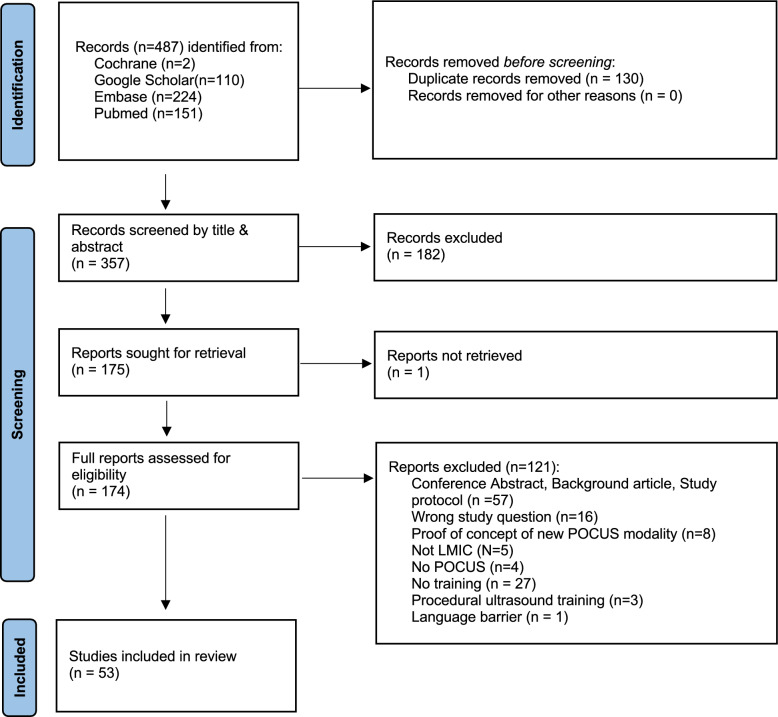
PRISMA Flow diagram of literature search and review: This flow chart illustrates the study selection process for this scoping review, following PRISMA guidelines. The initial literature search was conducted on December 8, 2022, with a search update on July 6, 2023. The diagram outlines the number of records identified, screened, and excluded at each stage, along with reasons for exclusion. It details the numbers of records removed due to duplication, non-relevant titles/abstracts, and other exclusion criteria, as well as the final number of studies included in the analysis

The database search yielded 357 unique publications. Following title and abstract screening 174 publications were retrieved for full text screening. A total of 121 records were excluded during full text screening and 53 publications were included for synthesis (Fig. 1 “PRISMA Flow diagram of literature search and review”) [24–76]. Most included studies were cross sectional (68%) without longitudinal follow-up and most studies collected data prospectively (87%); most publications employed quantitative methods (71%) (Supplement 3). Seventy percent of records were published within the past 5 years (between 2018 and 2023). All studies that met the inclusion criteria and were included in the review were published in English.

### Methodological quality of included studies

Of the 53 studies, nine studies (17%) were deemed to exhibit high methodological quality, 22 studies (42%) were rated as having moderate quality and 22 studies (42%) were evaluated as having limited methodological quality (Supplement 4, Supplement 5). All studies meeting the inclusion criteria were analysed, regardless of methodological quality. The primary limitation was inconsistently reported and missing data, which varied across studies and affected generalizability. No studies were excluded based on data completeness alone, but missing data points were noted. While methodological limitations existed, our focus on descriptive data rather than outcome comparisons minimizes their impact on findings.

### Study objectives

The publications included in this review had varying study objectives, that were grouped in three main categories: (i) studies focusing on training design (n = 27, 51%), i.e., training setup, practicalities, training curriculums and the feasibility of conducting POCUS trainings in a certain setting [24, 27–29, 31, 35, 37, 38, 43, 45, 47, 48, 51, 53, 55–60, 63, 68–71, 73, 76]; (ii) studies focusing on clinical utility (n = 7, 13.2%), i.e., appropriateness of POCUS or certain POCUS modalities in different, new or specific settings [25, 30, 39, 44, 52, 66, 74]; and (iii) studies evaluating short- and long-term POCUS competence and uptake (n = 19, 36%) [26, 32–34, 36, 40–42, 46, 49, 50, 54, 61, 62, 64, 65, 67, 72, 75]. Although some studies focused on clinical utility or evaluation of POCUS skills, all publications included in this review provided key facts on training designs.

### Geographical distribution

**Fig. 2:**
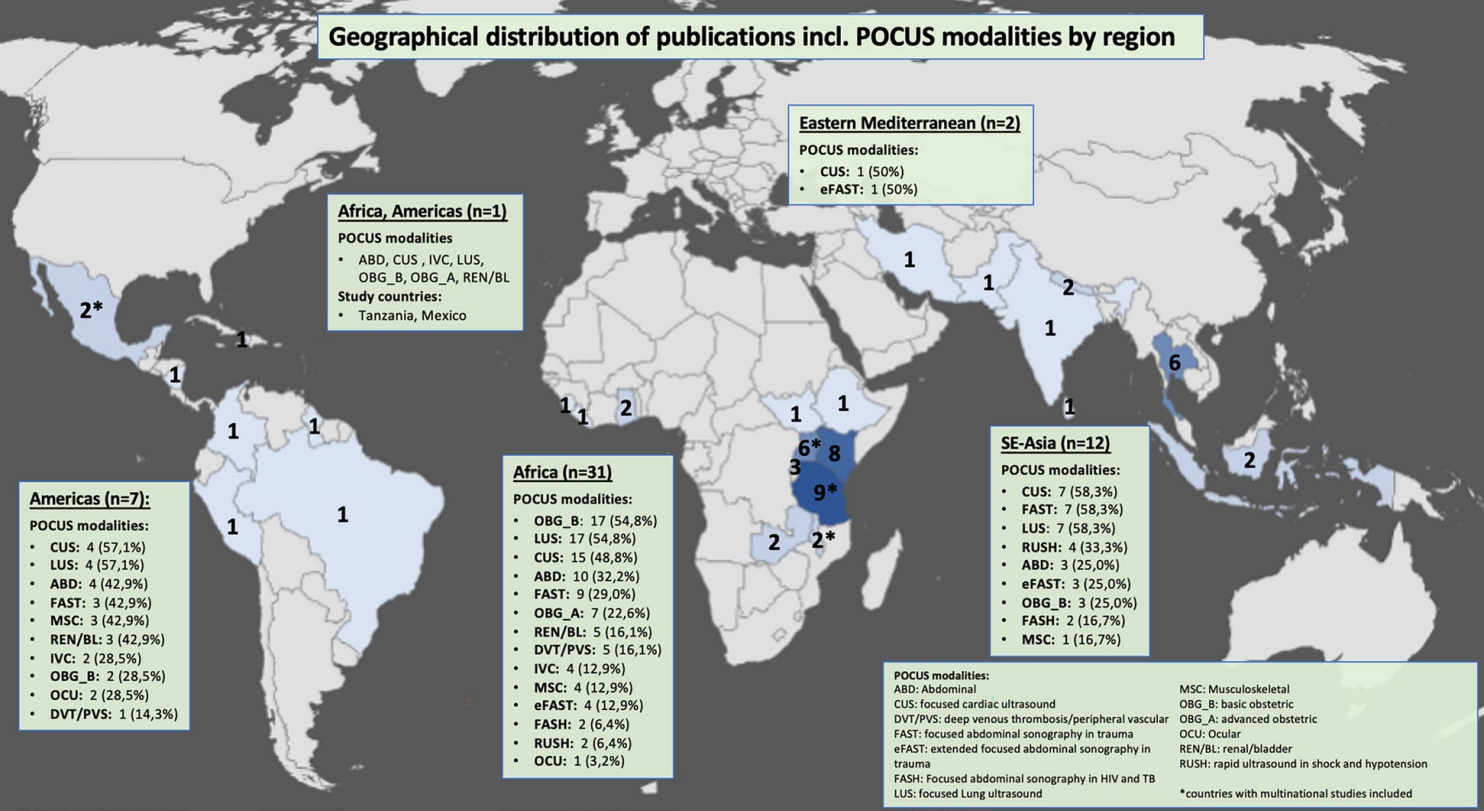
Geographical distribution of publications incl. POCUS modalities by region: Blue-shaded countries represent study locations, with darker shades indicating a higher number of studies conducted. Black numbers within each country denote the number of studies per country (note: asterisks* indicate countries with multinational studies included). Green boxes outline different WHO world regions, showing the total number of publications from each region (n) and the POCUS modalities reported as being taught within these regions (n = frequency, % = percentage of total publications from that region). One additional box ("Africa, Americas") represents a single intercontinental study conducted in both Tanzania (WHO African Region) and Mexico (WHO Region of the Americas), and is shown separately to reflect this cross-regional context

The majority of included publications (n = 31, 59%) originated from the African continent, while 12 (23%) studies were conducted in South East Asia, seven (13%) in the Americas, two (4%) in the Eastern Mediterranean Region and one (2%) study was a multinational study conducted in Africa and the Americas (Fig. 2; Table 1).

**Table 1:** Overview of included studies

First author Year of publication	Study design	Country of study	Clinical setting	Medical field	POCUS modality	No of trainees	Trainee profession	Training structure	Length of training	Training body	No of trainers
Barsky et al. [30]	C/P	Tanzania	Primary	n.a	FASE	6	STU	SC + MENT	≤ 1 week	n.a	n.a
Dreyfuss et al. [24]	L/P	Peru	Unclear	EM	CUS, LUS, FAST, ABD, MSC, REN/BL, IVC, OCU	3	MD	SC + MENT + RC	≤ 1 month (> 1 week)	local uni; intl uni	n.a
Bell et al. [31]	C/R	Kenya	Secondary	MED	OBG_B, eFAST	81	MD, RN, CO	SC + RC	≤ 1 week (> 1day)	local uni	n.a
Vanderburg et al. [38]	C/P	Sri Lanka	Tertiary	PUL	LUS	5	MD	SC + MENT	≤ 1 week (> 1day)	local uni	1
Shumbusho et al. [52]	C/P	Rwanda	Tertiary	SURG	LUS	15	MD	SC	≤ 1day	local uni	n.a
Hall et al. [37]	L/P	Tanzania	Primary & secondary	OBG	OBG_B, OBG_A	13	MD, MW, CO	SC + MENT	≤ 1 year (> 1 month)	iNGO	6
Wanjiku et al. [40]	C/P	Kenya	Primary	PC	OBG_B, CUS, FAST	33	MD, RN, CO	SC + RC	n.a	local uni	n.a
Shokoohi et al. [41]	C/P	Malawi, Tanzania, Uganda	Tertiary	OBG, PC, PED; MED, AN	OBG_B, OBG_A, CUS, LUS, ABD, MSC, REN/BL, OCU, DVT/PVS	63	MD, MW	SC + RC	n.a	intl uni	n.a
Reynolds et al. [73]	C/P	Tanzania, Mexico	Secondary & tertiary	EM	OBG_B, OBG_A, CUS, LUS, ABD, REN/BL, IVC	23	MD	SC	≤ 1 month (> 1 week)	intl uni	n.a
Vinayak et al. [69]	L/P	Kenya	Secondary	OBG	n.a	9	MW	SC + MENT	≤ 1 month (> 1 week)	local uni	n.a
Ienghong et al. [46]	C/R	Thailand	Tertiary	EM	CUS, LUS, RUSH, FAST, eFAST	8	MD	SC	≤ 1 month (> 1 week)	local uni	n.a
Ienghong et al. [45]	C/R	Thailand	Tertiary	EM	CUS, LUS, RUSH, FAST, eFAST	18	MD	SC	≤ 1 month (> 1 week)	local uni	n.a
Azizi et al. [53]	C/P	Pakistan	Tertiary	EM	eFAST	31	MD	SC	≤ 1day	local uni	n.a
Shah et al. [70]	L/P	Uganda	Primary & secondary	OBG	OBG_B, OBG_A	25	MD, MW, RN	SC + MENT	≤ 1 month (> 1 week)	local NGO	n.a
Aspler et al. [28]	C/P	Ethiopia	Tertiary	EM	LUS, FAST, eFAST, IVC	17	MD	LONG	n.a	intl uni	n.a
Lee et al. 2017[49]	C/P	Indonesia	Primary	PC	OBG_B, CUS, LUS, FAST, FASH	52	MD	SC	≤ 1 month (> 1 week)	n.a	n.a
Dornhofer et al. [54]	C/P	Indonesia	Primary	OBG, PC	OBG_B, CUS, LUS, FAST, ABD, FASH	55	MD, MW, RN	SC	≤ 1 month (> 1 week)	intl uni	8
Shaffer et al. 2017[62]	C/P	Tanzania	Tertiary	EM	CUS, LUS, FAST	8	MD	SC	≤ 1 week (> 1day)	intl uni	4
Bentley et al. [32]	L/P	Liberia	Secondary & Tertiary	OBG	OBG_B, OBG_A	31	MW	SC	≤ 1 week (> 1day)	intl uni	n.a
Shresta et al. [71]	C/P	Nepal	Primary & Secondary	EM, PC	OBG_B, CUS, LUS, RUSH, eFAST, ABD, MSC	50	MD	SC	≤ 1 week (> 1day)	intll uni	n.a
Marihan et al. [64]	C/P	Tanzania	Primary	n.a	OBG_B, CUS, LUS, FAST, ABD	150	STU	SC	≤ 1 month (> 1 week)	intl uni	6
Jones et al. [50]	C/P	Kenya	Tertiary	PC	OBG_B, CUS, LUS, RUSH, ABD, FASH, DVT/PVS	41	MD	SC	≤ 1 week (> 1day)	local uni, intl uni	n.a
Kimambo et al. [55]	C/P	Tanzania	Secondary	CARD	CUS	8	MD, CO	SC + MENT	≤ 1 week (> 1day)	local uni	3
Wanjikua et al. [39]	L/P	Kenya	Primary	EM	OBG_B, eFAST	14	MD, RN, CO, RT	LONG	n.a	intl uni	n.a
Nadimpalli et al. [25]	C/P	South Sudan	Primary & Secondary	PED	LUS	6	CO	SC + MENT	≤ 1 week (> 1day)	iNGO	1
Kimberley et al. [43]	L/P	Zambia	Secondary & Tertiary	OBG	OBG_B, OBG_A	21	MW	SC + MENT + RC	≤ 1 year (> 1 month)	intl uni	n.a
Henwood et al. [67]	L/P	Rwanda	Secondary & Tertiary	n.a	CUS, LUS, FAST, ABD, IVC	17	MD	LONG	≤ 1 year (> 1 month)	iNGO	n.a
Gómez Betancourt et al. [60]	C/P	Columbia	Tertiary	EM	IVC	8	MD	SC	≤ 1day	local uni	1
House et al. [35]	C/P	Nepal	Secondary & tertiary	EM	LUS	21	MD	SC + MENT	≤ 1 week (> 1day)	local uni	2
Rao et al. [76]	C/P	Haiti	Primary	PC	CUS	15	MD	SC	≤ 1 week (> 1day)	iNGO	n.a
Ienghong et al. [44]	L/R	Thailand	Tertiary	EM	CUS, RUSH, FAST	9	MD	SC	≤ 1 month (> 1 week)	local uni	n.a
Vanichkulbodee et al. [26]	C/P	Thailand	Tertiary	EM	FAST	234	STU	n.a	n.a	local uni	n.a
Kolbe et al. 2014 [56]	L/P	Nicaragua	Primary	NA	OBG_B, CUS, LUS, ABD, DVT/PVS	4	MD, RN	SC + MENT + RC	n.a	local NGO	n.a
Ienghong et al. [47]	C/R	Thailand	Tertiary	EM	n.a	18	MD	SC	≤ 1 month (> 1 week)	local uni	n.a
Sabatino et al. [75]	C/P	Sierra Leone	Secondary	EM	LUS, ABD, MSC, DVT/PVS	2	CO	SC + MENT + RC	≤ 1 month (> 1 week)	iNGO	4
Stolz et al. [59]	C/R	Uganda	Tertiary	EM	OBG_B, CUS, LUS, FAST, ABD, MSC, REN/BL, DVT/PVS	13	RN	LONG	> 1 year	n.a	n.a
Henwood et al. [66]	L/P	Rwanda	Primary & secondary	EM	OBG_B, CUS, LUS, ABD, REN/BL, IVC	n.a	MD	SC + MENT + RC	≤ 1 month (> 1 week)	n.a	n.a
Silva et al. [51]	C/P	Brazil	Unclear	MED	CUS, LUS, FAST, MSC	53	MD	SC	≤ 1 week (> 1day)	local uni	n.a
Terry et al. [29]	L/P	Uganda	Primary	EM	FAST	10	CO, STU	LONG	≤ 1 month (> 1 week)	n.a	n.a
Haldeman et al. [42]	L/P	Zambia	Primary	PC	OBG_B, CUS, LUS, RUSH, ABD, REN/BL, IVC, DVT/PVS	10	MD	LONG	≤ 1 year (> 1 month)	local uni	n.a
Osei-Ampofo et al. [61]	C/P	Ghana	Tertiary	EM	CUS, LUS	20	MD	SC	≤ 1 month (> 1 week)	local uni	n.a
Tafoya et al. [74]	C/P	Ghana	Tertiary	EM	CUS, LUS	20	MD	SC + MENT	≤ 1 month (> 1 week)	local uni	3
Dreizler et al. [58]	C/P	Kenya	Primary	PC	OBG_B, OBG_A, eFAST	6	MD, RN, CO, RT	MENT	≤ 1 year (> 1 month)	intl uni	n.a
Nazari et al. [36]	C/P	Iran	Secondary & tertiary	PED, CARD	CUS	7	MD	SC	≤ 1 week (> 1day)	local uni	2
Damgengkajornwong et al. [34]	C/P	Thailand	Tertiary	CARD	CUS	74	MD	SC	≤ 1day	local uni	n.a
Boniface et al. [57]	NA/R	Malawi, Tanzania, Uganda	Tertiary	n.a	CUS, LUS, FAST, ABD, FASH; REN/BL	63	MD, MW	SC + MENT + RC	≤ 1 week (> 1day)	intl uni	n.a
Rominger et al. [27]	L/P	Mexico	Primary	PC	OBG_B, LUS, FAST, ABD, MSC, REN/BL, OCU	8	MD	LONG	> 1 year	iNGO	6
Bhoi et al. [33]	C/P	India	Tertiary	TRAUM	FAST	5	MD	SC + MENT	≤ 1 week (> 1day)	local uni	n.a
Bui et al. [63]	C/P	Guyana	Secondary & Tertiary	URO	REN/BL	20	MD	SC + MENT	n.a	intl uni	n.a
Schmidt et al. [48]	C/P	Uganda	Tertiary	EM, PED	CUS, LUS	14	MD, CO	SC + MENT	n.a	intl uni	1
Vinayak et al. [68]	L/P	Kenya	Unclear	OBG	OBG_B	3	MW	SC + MENT	≤ 1 month (> 1 week)	local uni	n.a
Denny et al. [72]	C/P	Tanzania	Primary & secondary	n.a	OBG_B, CUS, LUS, FAST, ABD, MSC	354	MD, RN, CO, STU	SC	≤ 1 month (> 1 week)	intl uni	n.a
Matiang'i et al. [65]	C/P	Kenya	Primary	OBG	OBG_B, OBG_A	45	MW	MENT	n.a	n.a	n.a

Overview of included studies: this table provides a summary of studies in the review. Study Design: C (cross-sectional), L (longitudinal), P (prospective), R (retrospective), NA (not applicable). Medical Fields: Abbreviations include AN (Anesthesia), CARD (Cardiology), EM (Emergency Medicine), MED (Medicine), OBG (Obstetrics/Gynecology), PC (Primary Care), PED (Pediatrics), PUL (Pulmonology), SURG (Surgery), TRAUM (Traumatology), URO (Urology). POCUS Modalities Taught: ABD (Abdominal POCUS), CUS (Cardiac ultrasound), DVT/PVS (Deep Venous Thrombosis/Peripheral Vascular Sonography), FAST (Abdominal Sonography in Trauma), eFAST (Extended FAST), FASH (Sonography in HIV/TB), FASE (Sonography in Echinococcosis), IVC (Inferior Vena Cava Measurement) LUS (Lung ultrasound), MSC (Musculoskeletal), OBG_B (Basic Obstetric), OBG_A (Advanced Obstetric), OCU (Ocular), REN/BL (Renal/Bladder), RUSH (Rapid Ultrasound in Shock and Hypotension). Trainee Profession: CO (Clinical Officer), MD (Medical Doctor), MW (Midwife), RN (Nurse), RT (Radiographer), STU (Student). Training Structure: SC (Short Course), MENT (Mentoring/Supervision), RC (Repeat Courses), LONG (Longitudinal Training). Training Body: Intl uni (International University ± local partner), local uni (Local University), iNGO (International NGO ± local partner), local NGO (Local Non-Governmental Organization)

### Target clinical setting

**Fig. 3:**
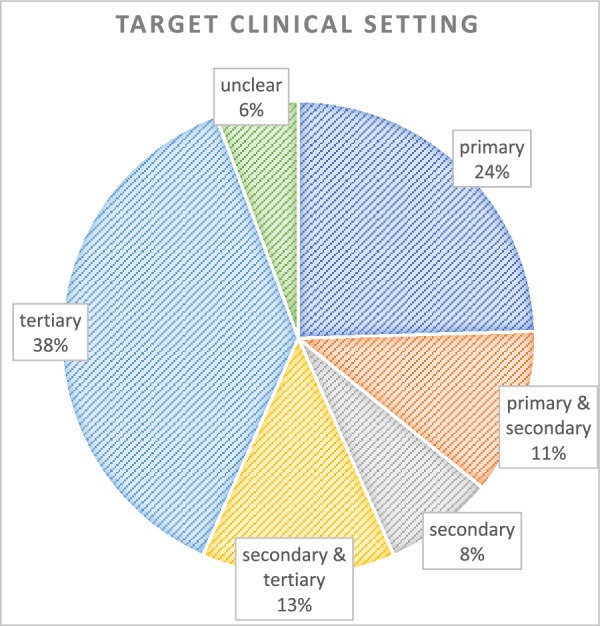
Target clinical setting distribution: The pie chart shows the distribution of target clinical settings across studies. Each sector represents one of the following categories: primary, primary & secondary, secondary, secondary & tertiary, tertiary, and unclear settings. Percentages indicate the proportion of studies within each category

Most studies (51%, n = 27) conducted POCUS training for a tertiary or mixed secondary and tertiary care setting, while 24% (n = 13) focused on a primary care setting (Fig. 3; Table 1).

### Trainee population

The trainee population was predominantly comprised of medical doctors, followed by clinical officers, midwifes, nurses, medical students and radiographers (Table 2). Although studies focusing solely on training professional radiographers were excluded, studies reporting the training of professional radiographers alongside bedside clinicians were included [39, 58]. Approximately half of the publications (n = 28, 53%) reported on the prior ultrasound skills of their trainees, which were predominantly basic, minimal, or absent. Trainees were commonly in the first five years of their professional careers (Table 3). The number of trainees per training ranged from two to 354 trainees, with median of 17 trainees per training (Table 1).

**Table 2:** Trainee population characteristics

Trainee population	No of publications	Median no per training (range)	Total no of trainees in all publications	References
Medical doctors	41 (77.4%)	12.5 (1–74)	622	[24, 27, 28, 31, 33–42, 44–58, 60–63, 66, 67, 70–74, 76]
Clinical officers	11 (20.7%)	3 (2–6)	28	[25, 29, 31, 37, 39, 40, 48, 55, 58, 72, 75]
Midwifes	10 (18.9%)	20 (3–45)	159	[32, 37, 41, 43, 54, 57, 65, 68–70]
Nurses	9 (17%)	4 (1–13)	37	[31, 39, 40, 54, 56, 58, 59, 70, 72]
Medical students	5 (9.4%)	150 (6–342)	740	[26, 29, 30, 64, 72]
Radiographers	2 (3.8%)	2 (1–4)	7	[39, 58]

Trainee population characteristics: the table displays information on trainee professions (medical doctors, clinical officers, midwives, nurses, medical students, radiographers) and their representation in the included studies. For each profession, the table provides: the number of publications (with corresponding percentages), median number of trainees per study (with range in brackets), total number of trainees across all publications (per subgroup), and references for studies involving training within each subgroup

**Table 3:** Trainees' Background Information

Trainees’ background	No (%) of publications	References
*Ultrasound experience*		
None	9 (17%)	[26, 34, 36, 38, 42, 51, 60, 69, 70]
Minimal	11 (20.7%)	[27, 31, 32, 37, 49, 50, 53–55, 67, 73]
Basic	2 (3.8%)	[30, 76]
Mixed	6 (11.3%)	[35, 46, 58, 62, 71, 74]
Not reported	25 (47.2%)	[24, 25, 28, 29, 33, 39–41, 43–45, 47, 48, 52, 56, 57, 59, 61, 63–66, 68, 72, 75]
*Avg. work experience (ys)*		
0	3 (5.7%)	[26, 38, 72]
1–5	13 (24.5%)	[28, 34, 35, 44, 45, 47, 52, 53, 60, 67, 68, 71, 74]
> 5	1 (1.9%)	[70]
Not reported	36 (67.9%)	
*Socioeconomic background*		
HIC	1 (1.9%)	[57]
LMIC	52 (98.1%)	

Trainees' background information: this table summarizes trainees' background details, including ultrasound experience level (none, minimal, basic, mixed, or not reported), average years of work experience (0; 1–5; > 5; or not reported), and socioeconomic background (HIC or LMIC). For each category, the table provides the number of publications (with corresponding percentages) and relevant references

All but two studies [46, 57] reported training of local personnel to provide POCUS services locally within their settings (Table 3). However, in one study trainees from an LMIC background were trained abroad (in another LMIC) to provide POCUS services in their home setting [46]. Additionally, one study reported training for high income country (HIC) trainees to subsequently provide POCUS in LMIC settings.

### Trainers and training bodies

Most POCUS trainings (n = 41, 77%) were held by universities or official training authorities with either a local (n = 24, 45%) or an international (n = 17, 32%) background (Table 4). Most trainers had an HIC or mixed background (35% and 11% of studies, respectively, 49% not reported). Only 2 publications (4%) reported an exclusively local training faculty [36, 38]. The training faculty predominantly comprised physician trainers (52% of studies) from diverse subspecialties with the majority being emergency physicians (n = 14, 50% of physician trainers) (Table 4). The trainer-to-trainee ratios ranged from 2:1 [75] up to 1:25 [64] with a median of 1:5.5 but were not consistently reported.

**Table 4:** Trainers' background and training bodies

Trainers’ background	No (%) of publications	References
*Geographical background*		
HIC	19 (35.8%)	[24, 27, 29, 31, 32, 37, 41, 48, 49, 54, 56–58, 61, 64, 72, 73, 75, 76]
Mixed (local & HIC)	6 (11.3%)	[28, 43, 62, 63, 70, 71]
Local	2 (3.8%)	[36, 38]
Not reported	26 (49%)	[25, 26, 30, 33–35, 39, 40, 42, 44–47, 50–53, 55, 59, 60, 65–69, 74]
*Professional background*		
Physician trainers	28 (52.8%)	[25, 27–29, 31, 32, 34, 36–38, 42, 44–48, 55–57, 59, 60, 62, 66, 67, 73–76]
– Emergency medicine specialist	14 (26.4%)	[27–29, 31, 32, 34, 37, 48, 57, 59, 62, 66, 67, 74]
– Emergency medicine resident	4 (7.5%)	[27, 28, 32, 62]
– Pediatrician specialist	4 (7.5%)	[25, 27, 36, 75]
– Radiology specialist	4 (7.5%)	[38, 66, 67, 75]
– Obstetrics specialist	3 (5.7%)	[37, 66, 67]
– Cardiology specialist	2 (3.8%)	[34, 55]
– Family Medicine specialist	1 (1.9%)	[62]
– Physician specialist (not specified)	9 (17%)	[42, 44–47, 56, 60, 73, 76]
Medical students	6 (11.3%)	[49, 51, 54, 64, 72, 74]
– First year medical students	3 (5.7%)	[49, 64, 72]
Ultrasound technicians	3 (5.7%)	[35, 55, 65]
Professional background not reported	18 (34%)	[24, 26, 30, 33, 40, 41, 43, 50, 52, 53, 55, 58, 61, 63, 68–71]
*Training body*		
Local university or official training body	24 (45.3%)	[24, 26, 31, 33–36, 38, 40, 42, 44–47, 50–53, 55, 60, 61, 68, 69, 74]
International university or official training body with local partner	17 (32.1%)	[24, 28, 32, 39, 41, 43, 48, 50, 54, 57, 58, 62–64, 71–73]
International NGO with local partner	6 (11.3%)	[25, 27, 37, 67, 75, 76]
Local NGO or initiative	2 (3.8%)	[56, 70]
Training body not reported	6 (11.3%)	[29, 30, 49, 59, 65, 66]

Trainers' background and training bodies: this table provides information on the background of trainers, including their geographical background (HIC, mixed local & HIC, local, or not reported) and professional background (physician trainers with subspecialties, medical students with first-year students as a subgroup, ultrasound technicians, or unspecified professional background). Additionally, the table outlines details on the training body, categorized as a local university or official training body, international university or official training body, international NGO with a local partner, local NGO or initiative, or unspecified training body. For each category, the table includes the number of publications (with corresponding percentages) and relevant references

### Length of training

**Fig. 4:**
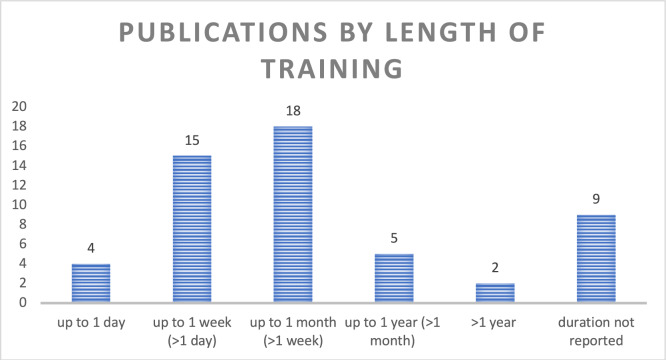
Number of publications by length of training this bar diagram displays the distribution of publications based on the length of training. Categories include: up to 1 day, up to 1 week (> 1 day), up to 1 month (> 1 week), up to 1 year (> 1 month), more than 1 year, and unspecified duration. The length of each bar represents the number of publications in each category, with the exact number displayed at the end of each bar

**Fig. 5:**
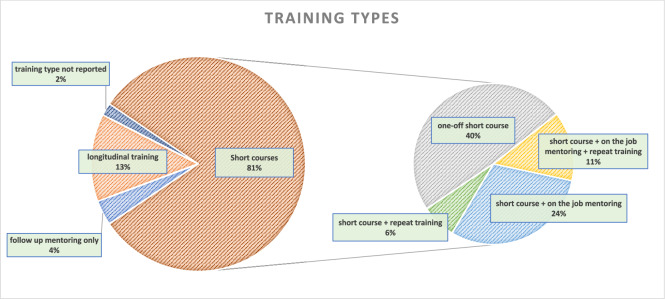
Distribution of training types: this pie-of-pie chart illustrates the distribution of different training types across studies. The left pie chart represents the main categories: short courses, longitudinal trainings, follow-up mentoring only, and unspecified training types. The right sub-pie chart further breaks down short course categories into: one-off short courses, short course with on-the-job mentoring and refresher training, short course with on-the-job mentoring, and short course with refresher training. Percentages in brackets represent the proportion of total publications, while segment sizes reflect sub-total percentages within each category

The duration of training programs varied significantly with some lasting only a few hours on a single day and others extending over a period of twelve months and more. The majority of programs were of a shorter duration of up to one month (Fig. 4; Table 1). Most publications (n = 43, 81%) described short courses, amongst which 40% (n = 17) were standalone sessions and 60% (n = 26) included some form of follow-up after the initial course. Seven publications (13%) reported on longitudinal trainings, featuring repeated sessions over an extended period and on-the-job mentoring, lasting from more than a month to over a year (Fig. 5).

All training-courses comprised theoretical didactic sessions followed by hands on training; 17 publications (32%) detailed the specific amounts of didactic and hands-on training with a varying but median theory-to-practice ratio of 1:3 [25, 27, 34, 35, 37, 46, 47, 49, 51, 54, 55, 62, 68, 69, 72, 73, 75].

### Ultrasound models and devices

The “models” for practical sessions included healthy volunteers, healthy pregnant volunteers, simulators, gelatine models (in cases where procedural ultrasound was part of the POCUS training) and patients. In general, most studies that reported on the type of ultrasound models (n = 34, 64%) integrated patients as ultrasound models (21/34, 62%) (Supplement 6).

A diverse range of ultrasound devices were utilized (Supplement 7). However, few studies (n = 21, 40%) documented the number of ultrasound devices utilized in trainings, with a median device-per-trainee ratio of 1:5.6 (Supplement 8). Additionally, actual scanning times per trainee were not consistently reported.

### Training content & POCUS modalities

Formal needs assessments, evaluating commonly prevalent pathologies and potentially applicable POCUS modalities, were only conducted before 23% (12/53) of trainings. [28, 31, 37, 39, 42, 47, 66, 67, 69, 70, 73, 75]. This proportion was fairly similar in publications with local training bodies such as local universities or local non-governmental organisations (NGOs) (6/26 23%) [31, 42, 47, 66, 69, 70] and those with an international training body such as international universities or international NGOs (6/23; 26%) [28, 37, 39, 67, 73, 75]

Twelve studies (23%) reported that the training was fully integrated in local training schemes or formal curricula [24, 26–29, 40, 42, 44, 45, 47, 59, 67], 4 studies (8%) had the training partly included in local training schemes [46, 65, 72, 74], whereas in 8 studies (15%) it was unclear if the training was integrated in local curricula or training schemes [31, 34, 36, 37, 52, 57, 58, 70]. Twenty-nine studies (55%) did not integrate the training in local training schemes.

Seven publications (13%) included a training of trainers’ course [24, 27, 28, 37, 41, 67, 70].

The majority of publications had a multi-disciplinary training content with a focus on one to two clinical specialties. The most common specialties were emergency medicine (n = 21, 40%), primary care (n = 10, 19%), obstetrics (n = 9, 17%), pediatrics (n = 4, 8%) and cardiology (n = 3, 6%) (Table 1).

Both organ focused and syndrome focused POCUS modalities were taught. The most common organ focused POCUS modalities were focused lung ultrasound (55%), focused cardiac ultrasound (53%) and basic obstetric ultrasound (43%). The most common syndrome focused protocols were focused abdominal sonography in trauma FAST (36%) and extended focused abdominal sonography in trauma eFAST (15%) (Table 5). The most commonly taught POCUS modalities by WHO region are presented in Supplement 8. In Africa basic obstetric ultrasound was the most commonly taught modality (17 studies). In South East Asia and in the Americas Region lung ultrasound as well as cardiac ultrasound were the most commonly taught modalities.

**Table 5:** POCUS modalities taught in trainings

POCUS type	POCUS modality	Description	No (%) of publications	References
Organ-focused POCUS	Basic obstetric POCUS	Transabdominal: confirmation of intrauterine pregnancy; detection of: twin pregnancy; placenta praevia; fetal hear beat; abnormal fetal presentation (breach)	23 (43.4%)	[27, 31, 32, 37, 39–43, 49, 50, 54, 56, 58, 59, 64–66, 68, 70–73]
	Advanced obstetric POCUS	Transabdominal or transvaginal: basic obstetric POCUS, fetal heart rate; placental location; gestational age (biparietal diameter, femur length)	8 (15.1%)	[32, 37, 41, 43, 58, 65, 70, 73]
	Focused cardiac ultrasound	Parasternal long & short axis; apical four chamber & subxiphoidal views (global RV & LV size & function, valve disasters, pericardial effusion); IVC (fluid status), pleural effusion	28 (52.8%)	[24, 34, 36, 40–42, 44–46, 48–51, 54–57, 59, 61, 62, 64, 66, 67, 71–74, 76]
	Focused lung ultrasound	Lung sliding, A-Lines, B-lines, pleural effusion, atelectasis, in some studies signs of pneumonia; often: BLUE protocol	29 (54.7%)	[24, 25, 27, 28, 35, 38, 41, 42, 45, 46, 48–52, 54, 56, 57, 59, 61, 62, 64, 66, 67, 71–75]
	Abdominal	Intraabdominal free fluid, kidneys (hydronephrosis, masses, stones), bladder (volume, urinary retention), liver (mass, abscess, cyst, hematoma), gall bladder (sludge, stones, cholecystitis), abdominal aorta, spleen (hematoma); ± intestines (bowel obstruction)	17 (32.1%)	[24, 27, 41, 42, 49, 50, 54, 56, 59, 64, 66, 67, 71–73, 75]
	Musculoskeletal/soft tissue	Abscess; joint effusion	8 (15.1%)	[24, 27, 41, 51, 59, 71, 72, 75]
	Renal/bladder	Hydronephrosis (± masses, stones), bladder volume, urinary retention, (± prostate)	9 (17%)	[24, 27, 41, 42, 57, 59, 63, 66, 73]
	Ocular	Detection of papilledema in patients with raised intracerebral pressure	3 (5.7%)	[24, 27, 41]
	IVC measurement	To evaluate volume status	7 (13.2%)	[24, 28, 42, 66, 67, 73]
	Peripheral vascular	Extremity deep venous thrombosis scan and peripheral arterial flow scan	6 (11.3%)	[41, 42, 50, 56, 59, 75]
Syndrome-focused POCUS	Rapid ultrasound for shock and hypotension (RUSH)	Heart (RV, pericardial effusion, LV function, VCI), Aorta, free intraabdominal fluid, Pneumothorax	6 (11.3%)	[42, 44–46, 50, 71]
	Focused assessment with sonography in trauma (FAST)	Detection of intraabdominal free fluid	19 (35.8%)	[24, 26–29, 33, 40, 44–46, 49, 51, 54, 57, 59, 62, 64, 67, 72]
	Extended focused assessment with sonography for trauma (eFAST)	FAST + Pleura Scan for pneumothorax (A Lines, Pleura gliding, M Mode: Sea shore/Barcode sign, Lung point)	8 (15.1%)	[28, 31, 39, 45, 46, 53, 58, 71]
	Focused abdominal ultrasound for HIV and TB (FASH)	Detection of pericardial effusion, pleural effusion & ascites ± presence of periportal/para-aortal lymph nodes, presence of focal liver or splenic lesions	4 (7.5%)	[49, 50, 54, 57]

POCUS modalities taught in trainings: this table summarizes the Point-of-Care Ultrasound (POCUS) modalities covered in training programs. The first column specifies the type of POCUS (organ-focused or syndrome-focused), the second column lists individual POCUS modalities, and the third column provides descriptions and relevant details for each modality. For each POCUS modality, the table includes the number of publications (with corresponding percentages) and associated references

### Training challenges

Thirty-three publications (62%) reported training challenges, while 20 publications (38%) did not report any difficulties conducting POCUS trainings [27, 33, 34, 36, 40, 42, 44, 46, 48, 50, 52, 53, 55, 59–61, 65, 69, 71, 75].

Training challenges classified into five principal categories: (i) resource constraints, (ii) educational limitations, (iii) operational challenges, (iv) (lack of) adaption to local context and (v) environmental and external factors (details in Table 6). A considerable number of training programs encountered comparable challenges, particularly in relation to resources and operational limitations.

**Table 6:** Training challenges

Category	Challenge	No (%) of publications	References	Comment
Resource constraints	*Internet* • Internet outages • Poor connectivity and coverage • Low speed • High cost of mobile data	7 (13.2%)	[39, 41, 47, 56–58, 72]	Resulting in limited uptake of online training materials as well as difficulties regarding timely image transfer and feedback as well as tele-mentoring sessions
	*Equipment problems*			
	• Low numbers of available ultrasound devices	4 (7.5%)	[26, 32, 45, 67]	Resulting in reduced scanning time per trainee
	• Security concerns	2 (3.8%)	[41, 57]	I.e., concerns for equipment theft, resulting in POCUS machines being “safely” locked away and not readily available at the bedside
	• Equipment malfunction, difficult and limited maintenance	6 (11.3%)	[37, 39, 56–58, 66]	„Two (of three) ultrasound machines had to be returned to the United States for repair during the training course (one with screen, the other with battery malfunction)”[37]
	• Relevant ultrasound equipment not available	2 (3.8%)	[32, 67]	No transvaginal probe available [32]
	• Lack of computers or mobile phones → prerequisite for tele-mentoring and for the use of electronic resources	2 (3.8%)	[25, 28]	Limited uptake of tele-mentoring and online resources
	*High cost* • Related to machine purchase, maintenance and image archiving	1 (1.9%)	[66]	Limiting scalability
Educational limitations	Limited training time	8 (15.1%)	[26, 41, 45, 57, 64, 70, 76]	Due to limited trainer time, due to competing clinical duties
	Limited availability of qualified trainers	4 (7.5%)	[24, 29, 37, 38]	Especially available locally – need for international trainers (more costly, time-constrained)
	Limited uptake of additional training sessions and supervised scans	2 (3.8%)	[37, 62]	Due to competing clinical duties
	Limited clinical supervision post training	1 (1.9%)	[74]	Due to limited trainer capacity
	Too many training applicants	2 (3.8%)	[64, 71]	Due to high volumes of applicants, not all applicants could be admitted to the training
	Training materials too comprehensive	1 (1.9%)	[31]	Training manual overwhelming and not concise enough for beginner trainees
	Difficulties finding pathology models with rare key pathologies	1 (1.9%)	[49]	Mostly healthy volunteer models used
	Training without real patients	1 (1.9%)	[47]	
	Lack of familiarity with computer handling	1 (1.9%)	[37]	Trainees unfamiliar with handling of keyboards, touch screens as well as track balls
	Eye fatigue	1 (1.9%)	[47]	Trainees unaccustomed to long screen times
Operational challenges	Competing clinical duties	5 (9.4%)	[39, 45, 51, 58, 70]	Due to high patient volumes and patient overcrowding at training hospitals, trainees sometimes had to switch between patient care and attending ultrasound trainings
	Personnel rotation	2 (3.8%)	[35, 67]	Due to personal reasons, planned trainee rotations
	Trainee dropout	1 (1.9%)	[37]	Due to either job transfer or lack of attendance
Environmental and external factors	Interruptions due to COVID 19 pandemic	3 (5.7%)	[28, 45, 63]	Limiting travel options for trainers and restricting normal training operations
	Power outages	3 (5.7%)	[37, 39, 56]	Limiting presentation options during lectures, scanning time as well as tele-mentoring options
	Extreme weather conditions	1 (1.9%)	[43]	Humidity, heat and dust challenging both human operator and ultrasound machine
Adaption to local context	Curriculum adjustments due to clinical needs at the target institution	3 (5.7%)	[30, 32, 68]	Bentley reported on a training on obstetric ultrasound with the initial curriculum including early pregnancy complications. As only late trimester patients presented to the target institution, the early pregnancy complications were removed from the curriculum. [32]
	Curriculum not comprehensive enough	2 (3.8%)	[25, 28]	Although multiple POCUS applications were suggested by trainees, only 3 (FAST, IVC, lung) were taught [28]
	Language Barriers	5 (9.4%)	[37, 54, 64, 72, 73]	Many trainings held in trainees’ second language
No training challenges reported	No training challenges reported	20 (37.7%)	[27, 33, 34, 36, 40, 42, 44, 46, 48, 50, 52, 53, 55, 59–61, 65, 69, 71, 75]	

Training challenges: this table presents the various challenges encountered in training programs. The first column lists challenge categories, while the second column details specific challenges within each category. The third column indicates the number of publications (with corresponding percentages), and the fourth column provides associated references. The final column includes comments offering further details on each reported challenge

Resource constraints encompassed three sub-categories: (i) Internet connectivity issues including frequent outages, poor coverage, slow speed and high cost. Those internet connectivity issues were hindering tele-mentoring, timely remote image review and feedback and limiting uptake of online training materials [39, 41, 47, 56–58, 72]; (ii) Equipment problems included limited availability of ultrasound devices [26, 32, 45, 67] and equipment (like probes) [32, 67], scarce local maintenance services [37, 39, 56–58, 66], and concerns regarding equipment theft [41, 57], limiting machine availability at the bedside for training. Hall et al. reported having to send 2/3 of their ultrasound machines to the United States for repairs during the training course, as no local repair option was available [37]. (iii) High cost was associated with equipment, maintenance and image archiving was another relevant resource constraint [66].

Educational limitations included limited training time [26, 41, 45, 57, 64, 70, 76] and trainer availability [24, 29, 37, 38], as well as issues regarding training design [47, 49, 74] and training manuals [31]. Additionally, Hall et al. reported that trainees had limited experience with computer handling, having rarely used trackballs, touchscreens or keyboards. This lack of familiarity with digital device interaction required additional time to accustom them to the equipment [37]. Similarly, Ienghong et al. noted that trainees experienced eye fatigue due to prolonged screen exposure, to which they were not accustomed, necessitating additional break times [47].

The primary operational challenges were competing clinical duties [39, 45, 51, 58, 70], personnel rotation [35, 67], and trainees’ attrition [37]. Environmental challenges included the impact of the coronavirus-19 pandemic [28, 45, 63], extreme weather conditions [43], and power outages [37, 39, 56]. Adaptation challenges involved language barriers [37, 54, 64, 72, 73] and curriculums that did not align with the local context [25, 28, 30, 32, 68].

### Included studies’ key findings

The majority of studies (n = 46; 87%) reported positive outcomes including an increase in knowledge, confidence, and POCUS skills or the feasibility of training. The overall outcome parameters across studies were numerous and varied, thereby precluding direct comparison of outcomes between studies. Positive outcomes comprised positive post training assessments with improvements in test scores and pass rates in practical examinations. The results of post course surveys indicated an increase in clinical confidence and an increase in ultrasound usage following the trainings [27, 50, 59, 62, 70, 71]. However, 7 (13%) publications reported inconclusive or negative results such as the inappropriateness of distance learning to acquire practical skills [39, 41, 47, 51, 55, 57, 64]. A detailed summary of reported outcomes is provided in Table 7.

**Table 7:** Outcome overview

Category	Outcome	No (%) of publications	References	Comment
Positive	Post training test improvement	N = 19 (36%)	[28, 32, 34, 37, 40, 42, 48, 49, 52–54, 62, 63, 68, 71, 72, 74, 76]	Demonstrated enhancement in trainees’ knowledge following training, typically measured through pre- and post-tests
	Adequate trainee-expert inter-operator agreement after training	N = 12 (23%)	[25, 29, 30, 33, 35, 36, 38, 43, 48, 52, 66, 75]	Trainees achieved sufficient agreement with expert ultrasound interpretations, indication skill acquisition and diagnostic reliability
	High post-training test or OSCE pass rates	N = 25 (47%)	[31, 32, 34, 37, 40, 42, 43, 46, 48–51, 53, 54, 61–63, 66–69, 72–75]	A majority of trainees successfully passed post-training assessments, such as written exams or objective structured clinical examinations (OSCEs)
	Increased clinical confidence and scan volume following training	N = 9 (17%)	[24, 27, 29, 36, 42, 43, 59, 66, 75]	Trainees performed more ultrasound scans after training, suggesting improved confidence and integration into clinical practice
	Positive trainee/trainer feedback	N = 21 (40%)	[26, 31, 32, 40, 42–45, 48–50, 54, 58, 62, 63, 65, 67, 70–73]	Trainers and trainees reported a favourable learning experience, including course structure, teaching quality, and perceived relevance
	Reported changes in patient management	N = 2 (4%)	[43, 56]	Training led to changes in clinical decision-making, such as altered diagnoses, treatment plans, or expedited referral
	Reduced examination time	N = 3 (6%)	[38, 60, 69]	Trainees demonstrated faster ultrasound examination times post-training, indicating improved efficiency and proficiency
Inconclusive	Mixed outcomes (both positive and negative results)	N = 3 (6%)	[47, 55, 64]	“No significant difference in scores between students taught in Kiswahili and English and those instructed only in English.” [64]
				“The 5-day training program was sufficient to train most clinicians to obtain basic cardiac images but not to accurately interpret them. “[55]
				“Participants can improve POCUS knowledge through virtual learning, but distant learning technique was not suitable for teaching practical skills “ [47]
	Limited reported outcomes (primarily program description)	N = 3 (6%)	[39, 41, 57]	Studies focused mainly on describing the training program rather than providing measurable training outcomes
Negative	Negative findings (regarding study question)	N = 1(2%)	[51]	“Substitution of eight hours of ultrasound simulation training for live model scanning in a 24-h training course did not enhance novice student performance on written and image acquisition tests”[51]

Outcome overview: this table summarizes the reported outcomes of POCUS training programs. The first column categorizes outcomes as positive, inconclusive, or negative. The second column details specific outcomes, including post-training test improvement, adequate trainee-expert inter-operator agreement, high post-training test or OSCE pass rates, increased clinical confidence and scan volume, positive trainee/trainer feedback, reported changes in patient management, reduced examination time, mixed results (both positive and negative), limited reported outcomes (primarily program descriptions), and negative findings regarding the study question. The third column provides the number of publications with percentages indicating the proportion of total included studies. The fourth column lists relevant references. The final column includes comments with explanations for the reported outcomes and supporting citations from the respective publications

### Best practice recommendations

The recommendations for best practice training outlined by the authors of the studies exhibited considerable variation. Some offered detailed guidance for future programs, while others provided no such guidance. Recommendations centred around four key themes: (i) training structure, (ii) trainers, (iii) training manuals, and (iv) ongoing supervision, feedback, and tele-mentoring (details in Table 8).

**Table 8:** Best practise recommendations

Theme	Recommendations	No (%) of publications	References
Training structure	• Short theoretical inputs with increased scanning times	4 (7.5%)	[70, 72, 74, 76]
	• Low trainer to trainee ratio	2 (3.8%)	[72, 70]
	• Protected time for training	1 (1.9%)	[58]
	• Online training	3 (5.7%)	[45–47]
Trainers	• Use of local trainers	1 (1.9%)	[70]
	• No improvements in outcomes with the use of translators during trainings observed	1 (1.9%)	[64]
Training manuals	• Written training manuals	1 (1.9%)	[70]
	• Online training materials the least useful	1 (1.9%)	[72]
	• Short & concise reading materials	1 (1.9%)	[31]
Ongoing Supervision, Feedback and Tele-mentoring	• Early OSCE exams during initial training	1 (1.9%)	[37]
	• Supervised scanning sessions	3 (1.9%)	[69, 70, 56]
	• Image review and feedback (tele-mentoring)	3 (1.9%	[24, 62, 69]
	• Online group sessions and teleconferences (tele-mentoring)	5 (1.9%)	[39, 24, 47, 56, 58]

*Best practice recommendations*: this table outlines the best practice recommendations proposed by authors across various publications. The first column lists the main categories, including training structure, trainers, training manuals, ongoing supervision, and tele-mentoring. The second column specifies individual recommendations within each category. The third column shows the number of publications (with corresponding percentages), and the fourth column provides relevant references

Key suggestions for training structure included maintaining brief and focused theoretical sessions and emphasizing hands-on scanning practice [70, 72, 74, 76], advocating for a low trainer-to-trainee ratio [70, 72], ensuring dedicated time for training [58], and incorporating online learning elements [45–47]. The preference for local trainers was highlighted as a means of enhancing language comprehension and contextual relevance [70]. However, no discernible benefits in outcomes were noted with the use of translators during sessions [64].

The recommendation for concise and straightforward printed training materials was also emphasized [31, 70].

To facilitate optimal learning and development, it was recommended that early evaluations [37], continuous supervision [56, 69, 70], and tele-mentoring with timely image review and feedback [24, 39, 47, 56, 58] as well as online group sessions and teleconferences [24, 39, 47, 56, 58] be employed in order to provide ongoing feedback and to efficiently distribute the limited availability of trainers to those trainees who would benefit the most from such support.

### Limitations reported by the authors

The majority of authors acknowledged the limitations of their studies, with only three publications (6%) failing to report any such limitations [57, 69, 70]. Some limitations were intrinsic to the study and training design. These included the use of small study cohorts (n = 24, 45%) [29–31, 35, 37–40, 42, 44, 45, 48, 49, 53, 58, 60, 61, 63, 64, 68, 71, 73, 75, 76], the lack of long term follow up (n = 10, 20%) [46, 49, 53, 54, 56, 63, 71–74], the exclusive reliance on a single study site (n = 8, 15%) [29, 35, 47, 52–54, 61, 63], the absence of a control group (n = 1, 2%) [43], the lack of blinding (n = 2, 4%) [32, 67], the absence of documentation of pre-course skills (n = 3, 6%) [49, 53, 74], and the use of incomplete evaluation methods (n = 5, 9%) [26, 27, 45, 62, 76]. Other limitations were related to insufficient adaptation to the local context, including the presence of language barriers (n = 5, 9%) [27, 30, 49, 51, 72] and the utilization of ultrasound protocols that were not specifically developed for the setting in question (n = 2, 4%) [25, 29].

Incomplete data emerged as a recurring issue in the execution of the studies (n = 7, 13%) [27, 29, 30, 40, 43, 58, 66].

The potential for bias related to trainee selection was a frequently discussed topic (n = 11, 21%) [33, 35, 48, 51, 53, 61, 63, 64, 67, 68, 74]. Many studies employed voluntary enrolment, which inherently selected for trainees with a pre-existing interest in ultrasound. Consequently, prior ultrasound experience was repeatedly identified as a potential confounder (n = 5, 9%) [42, 61, 67, 73, 76]. Studies that conducted surveys noted the possibility of recall bias, particularly in relation to the reported frequencies of ultrasound usage (n = 4, 8%) [26, 42, 45, 50] (details in Table 9). Limitations in training design and trainees’ participation echoed challenges previously reported in the “Training Challenges” section.

**Table 9:** Limitations reported by the authors

Category	Limitation reported by the authors	No (%) of publications	References
Study design	Small study cohort	24 (45.3%)	[29–31, 35, 37–40, 42, 44, 45, 48, 49, 53, 58, 60, 61, 63, 64, 68, 71, 73, 75, 76]
	Lack of long term follow up	10 (18.9%)	[46, 49, 53, 54, 56, 63, 71–74]
	Study performed at a single institution	8 (15.1%)	[29, 35, 47, 52–54, 61, 63]
	Impact on patient care/change in clinical management not evaluated	5 (9.4%)	[32, 35, 43, 71, 74]
	Incomplete evaluation method i.e., no OSCE, no clinical exam	5 (9.4%)	[26, 27, 45, 62, 76]
	Language barriers	5 (9.4%)	[27, 30, 49, 51, 72]
	Pre-course ultrasound skills not reported	3 (5.7%)	[49, 53, 74]
	No blinding	2 (3.8%)	[32, 67]
	Ultrasound protocol not developed for this setting	2 (3.8%)	[34, 35]
	No comparison to radiological gold standard	2 (3.8%)	[25, 29]
	No control group	1 (1.9%)	[43]
Study execution	Incomplete data	7 (13.2%)	[27, 29, 30, 40, 43, 58, 66]
Potential bias	Trainee selection as potential bias	11 (20.8)	[33, 35, 48, 51, 53, 61, 63, 64, 67, 68, 74]
	Previous ultrasound experience as possible confounder	5 (9.4%)	[42, 61, 67, 73, 76]
	Possible recall bias	4 (7.6%)	[26, 42, 45, 50]
	Grading for OSCE or image quality is a subjective measurement	3 (5.7%)	[42, 49, 63]
Training design	Healthy ultrasound models only	3 (5.7%)	[36, 49, 54]
	Low prevalence of key pathology	3 (5.7%)	[36, 38, 55]
	Limited number of trainers/equipment	2 (3.8%)	[41, 44]
	Short training period	2 (3.8%)	[55, 75]
	High cost	2 (3.8%)	[39, 59]
Participation issues	Participants drop out or turnover	3 (5.7%)	[27, 65, 66]
	Limited attendance to OSCE/tests/follow up	2 (3.8%)	[32, 51]
	Competing clinical responsibilities	2 (3.8%)	[33, 43]
	COVID 19	1 (1.9%)	[28]

Limitations reported by the authors: this table summarizes the limitations identified by authors across various publications. The first column lists the main limitation categories, including study design, study execution, potential bias, training design, and participation issues. The second column provides specific limitations within each category. For each limitation, the table includes the number of publications (with corresponding percentages) and relevant references

## Discussion

The findings of this scoping review shed light on the landscape of point-of-care ultrasound (POCUS) training programs focusing on low-resource settings. The availability of studies from various regions with limited diagnostic capacity underscores interest in and the potential of POCUS education to empower frontline healthcare providers with the necessary tools to improve patient care and outcomes. We identified a surprisingly high number of relevant publications, most of them prospective and rather recent.

Almost two thirds of publications originated from Africa, a quarter from Southeast-Asia and some from the Americas. The predominance of publications from the African continent aligns with Abrokwa et al.’s recent review on POCUS task shifting, which similarly reports a majority of studies originating from the African continent [6]. A possible reason for the strong representation of studies from Africa could be the shortage of radiological services and specialised sonographers, especially in rural areas of Africa compared to other World Health Organisation (WHO) regions [10, 11]. This aspect might limit the generalisability of findings to other world regions but underscores the importance of POCUS especially in the absence of other forms of imaging.

Most studies were conducted at tertiary or combined secondary/tertiary healthcare facilities, contrasting with the emphasised need for better diagnostics access at the primary care level in LMICs [8, 9, 11]. Almost half of the publications reported a local and a third of publications an international university or official training body. This could explain the strong focus on tertiary healthcare facilities as they are more likely linked to university structures that, as academic institutions, are inherently more inclined to research and publications. Strategies are needed to expand access to POCUS training, evaluation, and research, especially in lower-level healthcare settings where it is most needed.

### Localization & sustainability

Context adaptation and sustainability of POCUS training programs is key for effective long-term diagnostic capacity building. To provide locally relevant and sustainable POCUS training programs, (i) conducting formal needs assessments, (ii) focusing on integrating the POCUS training in local training schemes and (iii) involving local faculty as well as (iv) conducting training of trainers’ courses is paramount.

Conducting a formal needs assessment before initiating a POCUS training program is critical to determine adequate content of trainings [12]. However, merely a quarter of the studies identified in this review reported conducting a formal assessment evaluating clinical needs prior to the training. In addition to needs assessments by surveys or interviews of local healthcare providers, POCUS programs may be elaborated by weighing approaches considering disease prevalence, impact of POCUS on patient management, and difficulty versus ease of respective POCUS applications [77, 78]. A study from South Africa evaluating the adequacy of the ultrasound curriculum for emergency physicians at secondary level hospitals within the Western Cape Province, identified lung POCUS as the most important application, followed by musculoskeletal POCUS, cardiac POCUS and focused abdominal sonography in HIV and TB (FASH) [77]. Applying the weighing concept, the South African study determined eFAST followed by FASH as the most important POCUS applications in the local context as these scored highest on disease prevalence, disease impact and POCUS difficulty. A study from Malawi that applied the same weighing approach and comprising different clinical settings including tuberculosis (TB) and HIV/TB wards, identified FASH followed by cardiac POCUS as most important POCUS applications for the local setting [78]. The most frequently taught POCUS applications in studies reviewed were primarily related to emergency medicine and obstetrics. Lung and cardiac POCUS ranked highest, followed by basic obstetric POCUS and syndrome-focused applications such as FAST or eFAST; however, only four studies in this review included FASH. While the suitability of FASH should be evaluated individually for each study setting, given the high prevalence of TB and HIV across much of sub-Saharan Africa, FASH appears to be underutilized in training programs across these settings.

Integration of POCUS training into local training schemes or formal curricula can enhance the sustainability of POCUS initiatives, increasing the likelihood that these methods become established procedures in routine care. Yet only a minority of publications reported that POCUS training was either fully or partly integrated in local training schemes or formal curricula. Another barrier to needs-focused training can be the training faculty if it is not familiar with the local clinical context of the trainees [70]. In around one third of the studies in this review, the trainers came exclusively from HICs and most trainers were physician specialists, possibly less familiar with general medicine, that most health care providers in LMIC may deal with. A promising strategy for building sustained capacity is to implement training of trainers (ToT) programs. However, only seven publications in this review reported such approaches. Shah et al. [70] present an example of an innovative and feasible ToT approach: at a high-volume district hospital, a group of midwives received a two-week course in obstetric POCUS followed by clinical supervision. The most skilled and enthusiastic midwives were selected for a one-day ToT session to become “master trainers.” Subsequently, a second group of midwives from remote health clinics attended an intensive two-day obstetric POCUS course at the district hospital. These participants returned to their clinics with the master trainers, who provided clinical supervision and proctored scans within the local context. This approach ensured relevant training while minimizing disruptions to clinical services at the remote health centers.

### Training practicalities

Practical aspects of trainings varied widely across publications. Performing POCUS is a complex psychomotoric task that requires the simultaneous coordination of hands (for probe positioning, adjusting settings, and recording on the console), eyes, and brain (to conceptualize anatomy, evaluate POCUS images for quality and diagnostic interpretation) [37, 79]. To develop adequate scanning skills, sufficient training time is essential. However, most training courses were short, lasting up to one month, though duration varied widely from a few hours on a single day to programs extending beyond a year. Several publications highlighted a lack of sufficient training time as a common challenge.

In a typical supervised scanning session, one trainee performs the scan while the trainer stands nearby, and two additional trainees observe the screen from either side of the patient's bed. Any additional trainees may struggle to see the screen clearly and are unlikely to gain much benefit. Consistently Denny et al. recommend low trainer-to-trainee ratios [72] and Shah et al. suggest an optimal ratio of 1 trainer for every 4 trainees [70]. However, most studies reported higher trainer to trainee ratios ranging up to 1:25, with a median of 1:5.5. Although device-to-trainee ratios were inconsistently reported, the median was low with only 1 device for every 6 trainees. Under these conditions, it is unsurprising that trainees experience a lack of sufficient scanning and training time.

Reported theory-to-practice ratios varied widely, with a median ratio of 1:3. Some authors suggested shorter theoretical inputs with increased scanning times to ensure appropriate hands-on teaching [70, 72, 74, 76] while others advocated for increased use of online (pre-) training options to reserve in-person sessions for live scanning practice [45–47].

### Training challenges

Training challenges were reported by two thirds of studies. The main operational challenges were trainee dropout and competing clinical duties interfering with training attendance and uptake of supervised scanning sessions. Due to high patient volumes and staff shortages, trainees sometimes had to switch between patient care and attending ultrasound trainings. Dreizler et al. recommend assigning protected training times in order to ensure attendance to POCUS course and supervised scanning sessions [58].

Limited training time was as a recurring challenge. A number of methods to overcome this problem were observed: Some studies performed extended longitudinal trainings with re-occurring refresher sessions and ongoing supervision, while others reduced the overall number of trainees significantly to less than 5 trainees to ensure sufficient in depth-training. Another commonly used strategy was to limit the number of POCUS modalities to only one or two protocols. Each approach has its trade-offs and needs to be balanced: in-depth training allows for more thorough knowledge and skill transfer but is more costly per trainee and time intensive. Reducing the scope of POCUS modalities taught, will consequently limit the potential for clinical use. Although the optimal balance of trainee-to-trainer ratios, POCUS modalities taught, and the length of training is still uncertain, the principle of “less is more” in respect to content appears to be beneficial in making the most effective use of the available time.

Due to a lack of locally available trainers, many trainings involved tele-mentoring and remote image review to provide ongoing feedback and supervision to trainees. Tele-mentoring was identified as a promising method to overcome limited on site-trainer time and to ensure ongoing clinical supervision and remote image review facilitated timely feedback remotely from an experienced POCUS trainer to the bedside clinician in unclear cases. Furthermore, online group sessions and teleconferences offer the chance to discuss rare and important cases, and to address current clinical challenges. To achieve this, reliable internet connectivity and funding for data packages need to be provided.

Ultrasound training models were most commonly patients and volunteers (12/34, 35%), exclusively healthy volunteers (10/34, 29%) or exclusively patients (7/34, 21%). Training solely with healthy volunteers may prevent trainees from encountering relevant pathologies, while training exclusively with patients could lead to a lack of familiarity with physiological findings. A balanced approach using both physiological and pathological models may offer the most effective training.

### Best practice recommendations

Despite these challenges, the majority of studies reported positive outcomes, including improvements in knowledge, confidence, and POCUS skills among trainees. Best practice recommendations centered around four key themes: training structure, trainers, training manuals and ongoing supervision, feedback and tele-mentoring (Table 8). Authors emphasized the importance of maintaining brief theoretical sessions focusing on hands-on training, assigning training time protected from clinical duties and incorporating online learning elements and advocating for a low trainer-to-trainee ratio to enhance training effectiveness. Ideally, local trainers should be employed, as the use of translators was not found to be effective. It was recommended that training materials be in print, short and concise to facilitate effective course preparation and provide easy reference at the bedside. Furthermore, the recommendation for early and ongoing supervision and tele-mentoring highlights the need for continuous support and feedback to sustain the impact of POCUS training programs in the long term.

### Limitations

Several limitations should be considered when interpreting the findings of this review. The methodological quality of the included studies varied, with a significant proportion rated as moderate or limited. The main reason for limited methodological quality was missing and inconsistently reported data, which varied across studies. However, as this review focuses on descriptive syntheses rather than outcome comparisons, variations in study quality are not likely to significantly impact on conclusions. Additionally, the majority of studies were cross-sectional in nature, limiting the ability to draw causal conclusions about the effectiveness of POCUS training interventions. Future research should prioritize longitudinal studies with robust study designs to evaluate the long-term impact of POCUS training on clinical practice and patient outcomes.

## Conclusion

In conclusion, this scoping review provides valuable insights into current concepts and experiences of POCUS training programs in low-resource settings. Key aspects identified were context integration and focusing on local needs, trainer availability and suitability, durable equipment and maintenance and focus on hands-on training, and including patients with relevant pathology into the training. By addressing these challenges and leveraging best practice recommendations, training conceivers, policymakers and healthcare stakeholders can optimize POCUS education initiatives to enhance and scale up diagnostic capacity, improve patient care, and ultimately contribute to the achievement of universal health coverage in resource-limited environments.

## Supplementary Information

Additional file1 (DOCX 886 KB)

## Data Availability

The datasets used and/or analysed during the current study are available from the corresponding author on reasonable request.

## Abbreviations

POCUS: Point of care ultrasound
LMICs: Low- and middle-income countries
OSF: Open science framework
PCC method: Participants/population-concept-context method
HIC: High-income country
NGO: Non-governmental organisation
FAST: Focused abdominal sonography in trauma
eFAST: Extended focused abdominal sonography in trauma
WHO: World Health Organisation
FASH: Focused abdominal sonography in HIV and TB
TB: Tuberculosis
HIV: Human immune-deficiency virus
ToT: Training of trainers
