# Supplementary material for: Concepts for point-of-care ultrasound training in low resource settings: a scoping review
Source: Ultrasound J. 2025 May 15;17:24. doi: 10.1186/s13089-025-00427-3 (PMC12081813; doi:10.1186/s13089-025-00427-3)
Supplement: Supplementary file 1 — Additional file1 (DOCX 886 KB) [file 13089_2025_427_MOESM1_ESM.docx]

## **Supplementary materials**

### **Supplement 1 : Search stategy (PubMed)**

((POCUS) or (point of care ultrasound) or (bedside ultrasound) or (point of care sonography) or (bedside sonography) or (bedside echocardiography))

AND

((training) or (education) or (course))

AND

(((LMIC) or (low income country) or (middle income country) or (developing country) or (resource limited setting))

OR

((Afghanistan[tw]) OR (Albania[tw]) OR (Algeria[tw]) OR (American Samoa[tw]) OR (Angola[tw]) OR (Argentina[tw]) OR (Argentine Republic[tw]) OR (Armenia[tw]) OR (Azerbaijan[tw]) OR (Bangladesh[tw]) OR (Belarus[tw]) OR (Byelarus[tw]) OR (Belorussia[tw]) OR (Belize[tw]) OR (Benin[tw]) OR (Bhutan[tw]) OR (Bolivia[tw]) OR (Bosnia[tw]) OR (Botswana[tw]) OR (Brazil[tw]) OR (Bulgaria[tw]) OR (Burma[tw]) OR ("Burkina Faso"[tw]) OR (Burundi[tw]) OR ("Cabo Verde"[tw]) OR ("Cape verde"[tw]) OR (Cambodia[tw]) OR (Cameroon[tw]) OR ("Central African Republic"[tw]) OR (Chad[tw]) OR (China[tw]) OR (Colombia[tw]) OR (Comoros[tw]) OR (Comores[tw]) OR (Comoro[tw]) OR (Congo[tw]) OR ("Costa Rica"[tw]) OR ("Côte d'Ivoire"[tw]) OR (Cuba[tw]) OR (Djibouti[tw]) OR (Dominica[tw]) OR ("Dominican Republic"[tw]) OR (Ecuador[tw]) OR (Egypt[tw]) OR ("El Salvador"[tw]) OR (Eritrea[tw]) OR (Eswatini[tw]) OR (Ethiopia[tw]) OR (Fiji[tw]) OR (Gabon[tw]) OR (Gambia[tw]) OR (Gaza[tw]) OR ("Georgia Republic"[tw]) OR (Georgian[tw]) OR (Ghana[tw]) OR (Grenada[tw]) OR (Grenadines[tw]) OR (Guatemala[tw]) OR (Guinea[tw]) OR ("Guinea Bissau"[tw]) OR (Guyana[tw]) OR (Haiti[tw]) OR (Herzegovina[tw]) OR (Hercegovina[tw]) OR (Honduras[tw]) OR (India[tw]) OR (Indonesia[tw]) OR (Iran[tw]) OR (Iraq[tw]) OR (Jamaica[tw]) OR (Jordan[tw]) OR (Kazakhstan[tw]) OR (Kenya[tw]) OR (Kiribati[tw]) OR (Korea[tw]) OR (Kosovo[tw]) OR (Kyrgyz[tw]) OR (Kirghizia[tw]) OR (Kirghiz[tw]) OR (Kirgizstan[tw]) OR (Kyrgyzstan[tw]) OR ("Lao PDR"[tw]) OR (Laos[tw]) OR (Lebanon[tw]) OR (Lesotho[tw]) OR (Liberia[tw]) OR (Libya[tw]) OR (Macedonia[tw]) OR (Madagascar[tw]) OR (Malawi[tw]) OR (Malay[tw]) OR (Malaya[tw]) OR (Malaysia[tw]) OR (Maldives[tw]) OR (Mali[tw]) OR ("Marshall Islands"[tw]) OR (Mauritania[tw]) OR (Mauritius[tw]) OR (Mexico[tw]) OR (Micronesia[tw]) OR (Moldova[tw]) OR (Mongolia[tw]) OR (Montenegro[tw]) OR (Morocco[tw]) OR (Mozambique[tw]) OR (Myanmar[tw]) OR (Namibia[tw]) OR (Nauru[tw]) OR (Nepal[tw]) OR (Nicaragua[tw]) OR (Niger[tw]) OR (Nigeria [tw]) OR (Pakistan[tw]) OR (Palau[tw]) OR (Panama[tw]) OR ("Papua New Guinea"[tw]) OR (Paraguay[tw]) OR (Peru [tw]) OR (Philippines[tw]) OR (Phillippines[tw]) OR (Philipines[tw]) OR (Phillipines[tw]) OR (Principe[tw]) OR (Rwanda[tw]) OR (Ruanda[tw]) OR (Samoa[tw]) OR ("Sao Tome"[tw]) OR (Senegal[tw]) OR (Serbia[tw]) OR ("Sierra Leone"[tw]) OR ("Solomon Islands"[tw]) OR (Somalia[tw]) OR ("South Africa"[tw]) OR ("South Sudan"[tw]) OR ("Sri Lanka"[tw]) OR ("St Lucia"[tw]) OR ("St Vincent"[tw]) OR (Sudan[tw]) OR (Surinam[tw]) OR (Suriname[tw]) OR (Swaziland[tw]) OR (Syria[tw]) OR ("Syrian Arab Republic"[tw]) OR (Tajikistan[tw]) OR (Tadzhikistan[tw]) OR (Tadjikistan[tw]) OR (Tadzhik[tw]) OR (Tanzania[tw]) OR (Thailand[tw]) OR (Timor[tw]) OR (Togo[tw]) OR (Tonga[tw]) OR (Tunisia[tw]) OR (Turkey[tw]) OR (Turkmen[tw]) OR (Turkmenistan[tw]) OR (Tuvalu[tw]) OR (Uganda[tw]) OR (Ukraine[tw]) OR (Uzbek[tw]) OR (Uzbekistan[tw]) OR (Vanuatu[tw]) OR (Venezuela[tw]) OR (Vietnam[tw]) OR ("West Bank"[tw]) OR (Yemen[tw]) OR (Zambia[tw]) OR (Zimbabwe[tw])))

### **Supplement 2: Methodological Quality of Included Studies**

5 question critical appraisal tool:

Q1 Was the pre-training skill and knowledge level of training participants reported? (e.g. prior US knowledge, formal training level, clinical experience)?

Q2 Was the post-training skill and knowledge level of training participants reported?

Q3 Was the evaluation method adequate & relevant to the training?

Q4 Were relevant input & process factors reported? (e.g. trainer/trainee ratio, no of training devices, training duration)

Q5 Are the acquired knowledge & skills relevant and applicable to the trainees’ daily work?

Each question to be answered with yes / partly / no with different numerical scores for each answer (yes=2 points, partly 1 point, no =0 points).

Scores 9-10 were considered high methodological quality, 7-8 moderate methodological quality, and 6 points and below were considered limited methodological quality.

The critical appraisal tool was inspired by JBI’s critical appraisal tools, especially the Checklist for analytical cross sectional studies [21] and Downs and Black’s checklists

### **Supplement 3: Study Design of Included Studies**

36 publications were cross sectional studies [24, 25, 27, 29, 30, 32-35, 37, 39, 40, 44-48, 50-54, 57-64, 70-75], 16 studies were longitudinal [23, 26, 28, 31, 36, 38, 41-43, 49, 55, 65-69]; one study could not be categorized as either cross sectional or longitudinal [56]. 46 had a prospective design [23-29, 31-42, 47-55, 57, 59-75], 7 were retrospective [30, 43-46, 56, 79]. 38 studies used quantitative methods [23, 24, 27-33, 35-40, 42-47, 49, 51, 53-55, 58, 59, 61-63, 65, 67, 68, 71, 73-75], 14 studies mixed methods [25, 26, 34, 41, 48, 50, 52, 57, 60, 64, 66, 69, 70, 72] and one study could not be categorized in either category [56].

### **Supplement 3Table Study designs of included Publications**

| **Study design** | **No. (%) of studies** | **References** |
| --- | --- | --- |
| **cross-sectional** | **36** (67.9%) | [24, 25, 27, 29, 30, 32-35, 37, 39, 40, 44-48, 50-54, 57-64, 70-75] |
| Prospective data collection | **31** (58.5%) |  |
| - mixed methods | 10 (18.9%) | [25, 34, 48, 50, 52, 57, 60, 64, 70, 72] |
| - quantitative | 21 (39.6%) | [24, 27, 29, 32, 33, 35, 37, 39, 40, 47, 51, 53, 54, 59, 61-63, 71, 73-75] |
| Retrospective data collection | **5** (9.4%) |  |
| - quantitative | 5 **(**9.4%) | [30, 44-46, 58] |
| **longitudinal** | **16** (30.2%) | [23, 26, 28, 31, 36, 38, 41-43, 49, 55, 65-69] |
| prospective data collection | **15** (28.3%) |  |
| - mixed methods | 4 (7.5%) | [26, 41, 66, 69] |
| - quantitative | 11 (20.8%) | [23, 28, 31, 36, 38, 42, 49, 55, 65, 67, 68] |
| Retrospective data collection | **1** (1.9%) |  |
| - quantitative | 1 (1.9%) | [43] |
| **not applicable** | **1** (1.9%) | [56] |
| Retrospective data collection | **1** (1.9%) | [56] |
| - not applicable | 1 (1.9%) | [56] |

Study designs of Included Publications: This table provides an overview of the study designs used in the included publications. The first column categorizes studies as cross-sectional, longitudinal, or not applicable, with subcategories for prospective and retrospective data collection. These are further sub-categorized by method type: mixed methods, quantitative methods, or not applicable. For each category, the table includes the number of publications (with corresponding percentages) and relevant references.

### **Supplement 4: Table S4 Methodological Quality of Included Studies (by rating)**

| **Methodological quality** | **Rating (numerical)** | **No (%) of studies** | **References** |
| --- | --- | --- | --- |
| High | 10 | 2 (3.8%) | [47, 54] |
|  | 9 | 7 (13.2%) | [33, 34, 36, 46, 49, 61, 69] |
| moderate | 8 | 14 (26.4%) | [29-31, 35, 37, 41, 45, 48, 50, 60, 66, 67, 72, 73] |
|  | 7 | 8 (15.1%) | [26, 27, 38, 43, 44, 53, 59, 74] |
| limited | 6 | 9 (17%) | [32, 39, 42, 51, 52, 55, 62, 63, 68] |
|  | 5 | 7 (13.2%) | [24, 25, 28, 65, 70, 71, 75] |
|  | 4 | 2 (3.8%) | [23, 55, 64] |
|  | 3 | 2 (3.8%) | [56, 57] |
|  | 2 | 2 (3.8%) | [40, 58] |

Table Methodological quality of included studies (by rating): This table presents an overview of the methodological quality ratings for the included studies. The first column categorizes studies by quality rating (high, moderate, limited), while the second column provides the corresponding numerical rating (ranging from 10 to 2). For each numerical rating, the table includes the number of publications (with corresponding percentages) and relevant references.

### **Supplement 5: Table S5 Methodological Quality of Included Studies by rating question**

| Category | Rating | No (percentage) of studies | References |
| --- | --- | --- | --- |
| Pre training knowledge & skills reported | Sufficiently | 19 (35.8%) | [25, 27, 29, 33-35, 37, 41, 46, 47, 49, 53, 54, 59, 66, 67, 69, 72, 73] |
|  | Partly | 19 (35.8%) | [26, 30-32, 36, 38, 39, 43-45, 48, 50-52, 60, 61, 63, 70, 75] |
|  | Not sufficiently | 15 (28.3%) | [23, 24, 28, 40, 42, 55-58, 62, 64, 65, 68, 71, 74] |
| Post training knowledge and skills reported | Sufficiently | 23 (43.4%) | [27, 29, 33, 34, 36, 39, 41, 45-49, 53, 54, 59-61, 67, 69-73] |
|  | Partly | 19 (35.8%) | [23, 24, 26, 28, 30-32, 35, 37, 42, 50, 52, 62, 64-66, 68, 74, 75] |
|  | Not sufficiently | 11 (20.8%) | [25, 38, 40, 43, 44, 51, 55-58, 63] |
| Evaluation methods adequate and relevant to the training | yes | 27 (51.1%) | [30, 31, 33-38, 42-44, 46-51, 54, 55, 60-63, 65, 66, 69, 74] |
|  | partly | 19 (35.8%) | [24, 26, 28, 29, 32, 39, 41, 45, 52, 53, 57, 64, 67, 68, 70-73, 75] |
|  | No | 7 (13.2%) | [23, 25, 58] [27, 40, 55, 56, 59] |
|  |  |  |  |
| Input and process factors reported(factors like No. of trainers, duration of training, No. of Ultrasound devices) | Sufficiently | 17 (32.1%) | [26, 30, 31, 36-38, 43-45, 47, 50, 54, 55, 59, 61, 68, 74] |
|  | Partly | 31 (58.5%) | [23-25, 27-29, 32-35, 40-42, 46, 48, 49, 51-53, 56, 57, 60, 62, 63, 66, 67, 69, 71-73, 75] |
|  | Not sufficiently | 5 (9.4%) | [39, 58, 64, 65, 70] |
| Acquired skills and knowledge relevant and applicable to trainees’ daily work | Yes | 45 (84.9%) |  |
|  | Partly | 8 (15%) | [37, 40, 53, 57, 59, 70, 71, 75] |

Table Methodological Quality of Included Studies by Rating Question: This table provides an overview of the methodological quality based on specific rating questions. The first column lists each rating category, while the second column shows possible responses for each question. For each response, the table includes the number of publications (with corresponding percentages) and relevant references.

**Supplement 6 Table Ultrasound Models Used in Trainings**

| **Ultrasound model** | **No. (subtotal%) of studies** | **References** |
| --- | --- | --- |
| US model reported | 34 |  |
| Healthy volunteers only | 10 (29.4%) | [28, 30, 39, 48, 53, 59, 63, 64, 71, 75] |
| healthy volunteers & simulator | 2 (5.9%) | [50, 52] |
| healthy volunteers & gelatine model | 1 (2.9%) | [70] |
| Patients only | 7 (20.6%) | [23, 32, 35, 41, 43, 57, 60] |
| Patients & simulator | 2 (5.9%) | [44, 46] |
| Patients & healthy volunteers | 12 (35.3%) | [24, 26, 31, 34, 36, 37, 56, 61, 66, 69, 72, 73] |
| US model not reported | 19 | [25, 27, 29, 33, 38, 40, 42, 45, 47, 49, 51, 54, 55, 58, 62, 65, 67, 68, 74] |
| Ultrasound models in obstetric POCUS: |  |  |
| Ultrasound models in basic obstetric pocus | 23 |  |
| - Patients | 7 | [26, 31, 36, 41, 57, 69, 72] |
| - healthy volunteers only (pregnancy status not stated) | 6 | [30, 48, 53, 63, 70, 71] |
| - Healthy pregnant volunteers only | 3 | [39, 64, 69] |
| Ultrasound models in advanced obstetric pocus | 8 |  |
| - Patients | 5 | [31, 36, 57, 69, 72] |
| - Healthy pregnant volunteers only | 1 | [64] |
| - Ultrasound model not reported | 2 | [40, 42] |

Table Ultrasound models Used in Trainings: This table provides details on the different ultrasound models utilized in training, categorized as: healthy volunteers only, healthy volunteers & simulator, healthy volunteers & gelatine model, patients only, patients & simulator, patients & healthy volunteers, and unspecified models. For each category, the table includes the number of publications (with corresponding subtotal percentages) and relevant references. Additionally, the table outlines POCUS models used in basic obstetric ultrasound training (patients, healthy volunteers only [pregnancy status unspecified], healthy pregnant volunteers only) and advanced obstetric ultrasound training (patients, healthy pregnant volunteers only, unspecified model). Each category lists the number of publications and associated references.

### **Supplement 7: Table Ultrasound Devices Used in Trainings**

| **Ultrasound device** | **No (percentage) of studies** | **References** |
| --- | --- | --- |
| SonoSite M-Turbo | 7 (13.2%) | [34, 36, 44, 45, 47, 51, 58] |
| VScan (GE) | 5 (9.4%) | [40, 54, 56, 61, 73] |
| Butterfly IQ | 5 (9.4%) | [41, 43, 44, 62, 63] |
| SonoSite NanoMaxx | 5 (9.4%) | [26, 29, 48, 71, 72] |
| Mindray M9 | 3 (5.7%) | [44, 45, 53] |
| SonoSite 180 | 2 (3.8%) | [31, 42] |
| SonoSim high fidelity simulator | 2 (3.8%) | [50, 52] |
| Esaote MyLab Alpha | 1 (1.9%) | [74] |
| GE Healthcare LOGIQ P6 | 1 (1.9%) | [33] |
| Mindray M7 | 1 (1.9%) | [53] |
| Philips IU22 | 1 (1.9%) | [25] |
| Philips Lumify | 1 (1.9%) | [24] |
| Philips VISIQ | 1 (1.9%) | [67] |
| Samsung EK07 | 1 (1.9%) | [35] |
| SonoSite Edge | 1 (1.9%) | [59] |
| SonoSite Edge 2 | 1 (1.9%) | [37] |
| SonoSite S Series | 1 (1.9%) | [50] |
| SonoSite Titan | 1 (1.9%) | [55] |

Table Ultrasound devices used in trainings: This table lists the different types of ultrasound devices utilized in training programs, as detailed in the first column. For each device type, the table provides the number of publications (with corresponding percentages) and relevant references.

### **Supplement 8: Table No of Ultrasound Devices per Training**

| No. of devices | No. (percentage) of studies | References |
| --- | --- | --- |
| 1 | 9 (17.0%) | [25, 29, 31, 37, 43, 51, 55, 59, 74] |
| 2 | 3 (5.7%) | [45, 58, 73] |
| 3 | 4 (7.5%) | [36, 41, 44, 61] |
| 4 | 3 (5.7%) | [26, 42, 50] |
| 6 | 1 (1.9%) | [63] |
| 16 | 1 (1.9%) | [56] |

Table No. of ultrasound devices per training: This table displays the number of ultrasound devices used per training session, as indicated in the first column. For each device count, the table provides the corresponding number of publications (with percentages) and relevant references.

### **Supplement 9: Table POCUS Modalities by WHO Region**

| **WHO Region** | **POCUS modality** | **No. of studies (% within WHO region)** | **References** |
| --- | --- | --- | --- |
| Africa (total: 31 studies) | Obstetric POCUS (basic-transabdominal) | 17 (54.8%) | [30, 31, 36, 38-42, 49, 57, 58, 63-65, 67, 69, 71] |
|  | Obstetric POCUS advanced (transabdominal or transvaginal) | 7 (22.6%) | [31, 36, 40, 42, 57, 64, 69] |
|  | Focused cardiac ultrasound | 15 (48.4%) | [39-41, 47, 49, 54, 56, 58, 60, 61, 63, 65, 66, 71, 73] |
|  | Focused lung ultrasound | 17(54.8%) | [24, 27, 40, 41, 47, 49, 51, 56, 58, 60, 61, 63, 65, 66, 71, 73, 74] |
|  | Abdominal POCUS | 10 (32.2%) | [40, 41, 49, 56, 58, 63, 65, 66, 71, 74] |
|  | Musculoskeletal/soft tissue | 4 (12.9%) | [40, 58, 71, 74] |
|  | Renal/bladder | 5 (16.1%) | [40, 41, 56, 58, 65] |
|  | Ocular | 1 (3.2%) | [40] |
|  | IVC measurement | 4 (12.9%) | [27, 41, 65, 66] |
|  | Peripheral vascular | 5 (16.1%) | [40, 41, 49, 58, 74] |
|  | Rapid ultrasound for shock and hypotension (RUSH) | 2 (6.4%) | [41, 49] |
|  | Focused assessment with sonography in trauma (FAST) | 9 (29%) | [27, 28, 39, 56, 58, 61, 63, 66, 71] |
|  | Extended focused assessment with sonography for trauma (eFAST) | 4 (12.9%) | [27, 30, 38, 57] |
|  | Focused abdominal ultrasound for HIV and TB (FASH) | 2 (6.4%) | [49, 56] |
| Africa, Americas (total:1 study) | Obstetric POCUS (basic-transabdominal) | 1 (100%) | [72] |
|  | Obstetric POCUS advanced (transabdominal or transvaginal) | 1 (100%) | [72] |
|  | Focused cardiac ultrasound | 1 (100%) | [72] |
|  | Focused lung ultrasound | 1 (100%) | [72] |
|  | Abdominal POCUS | 1 (100%) | [72] |
|  | Renal/bladder | 1 (100%) | [72] |
|  | IVC measurement | 1 (100%) | [72] |
| Americas (total: 7 studies) | Obstetric POCUS (basic-transabdominal) | 2 (28.5%) | [26, 55] |
|  | Focused cardiac ultrasound | 4 (57.1%) | [23, 50, 55, 75] |
|  | Focused lung ultrasound | 4 (57.1%) | [23, 26, 50, 55] |
|  | Abdominal POCUS | 3 (42.9%) | [23, 26, 55] |
|  | Musculoskeletal/soft tissue | 3 (42.9%) | [23, 26, 50] |
|  | Renal/bladder | 3 (42.9%) | [23, 26, 62] |
|  | Ocular | 2 (28.5%) | [23, 26] |
|  | IVC measurement | 2 (28.5%) | [23, 59] |
|  | Peripheral vascular | 1 (14.3%) | [55] |
|  | Focused assessment with sonography in trauma (FAST) | 3 (42.9%) | [23, 26, 50] |
|  |  |  |  |
| Eastern Mediterranean (total:2 studies) | Focused cardiac ultrasound | 1 (50.0%) | [35] |
|  | Extended focused assessment with sonography for trauma (eFAST) | 1 (50.0%) | [52] |
| South East Asia (total:12 studies) | Obstetric POCUS (basic-transabdominal) | 3 (25.0%) | [48, 53, 70] |
|  | Focused cardiac ultrasound | 7 (58.3%) | [33, 43-45, 48, 53, 70] |
|  | Focused lung ultrasound | 7 (58.3%) | [34, 37, 44, 45, 48, 53, 70] |
|  | Abdominal POCUS | 3 (25.0%) | [48, 53, 70] |
|  | Musculoskeletal/soft tissue | 1 (8.3%) | [70] |
|  | Rapid ultrasound for shock and hypotension (RUSH) | 4 (33.3%) | [43-45, 70] |
|  | Focused assessment with sonography in trauma (FAST) | 7 (58.3%) | [25, 32, 43-45, 48, 53] |
|  | Extended focused assessment with sonography for trauma (eFAST) | 3 (25.0%) | [44, 45, 70] |
|  | Focused abdominal ultrasound for HIV and TB (FASH) | 2 (16.7%) | [48, 53] |

Table POCUS modalities by WHO Region: This table details the types of POCUS modalities taught across different WHO regions. The first column lists each WHO region, with the total number of studies from that region indicated in brackets. The second column specifies the POCUS modalities. The third column shows the actual number of studies (n), with the percentage of the regional subtotal in brackets. The fourth column includes relevant references.
